# Idiopathic intracranial hypertension presenting as CSF rhinorrhea

**DOI:** 10.4103/0972-2327.61286

**Published:** 2010

**Authors:** K. Saifudheen, Abdul Gafoor, G. Arun, P. Abdurahiman, James Jose

**Affiliations:** Department of Neurology, Medical College, Calicut-8, Kerala, India

**Figure 1:**
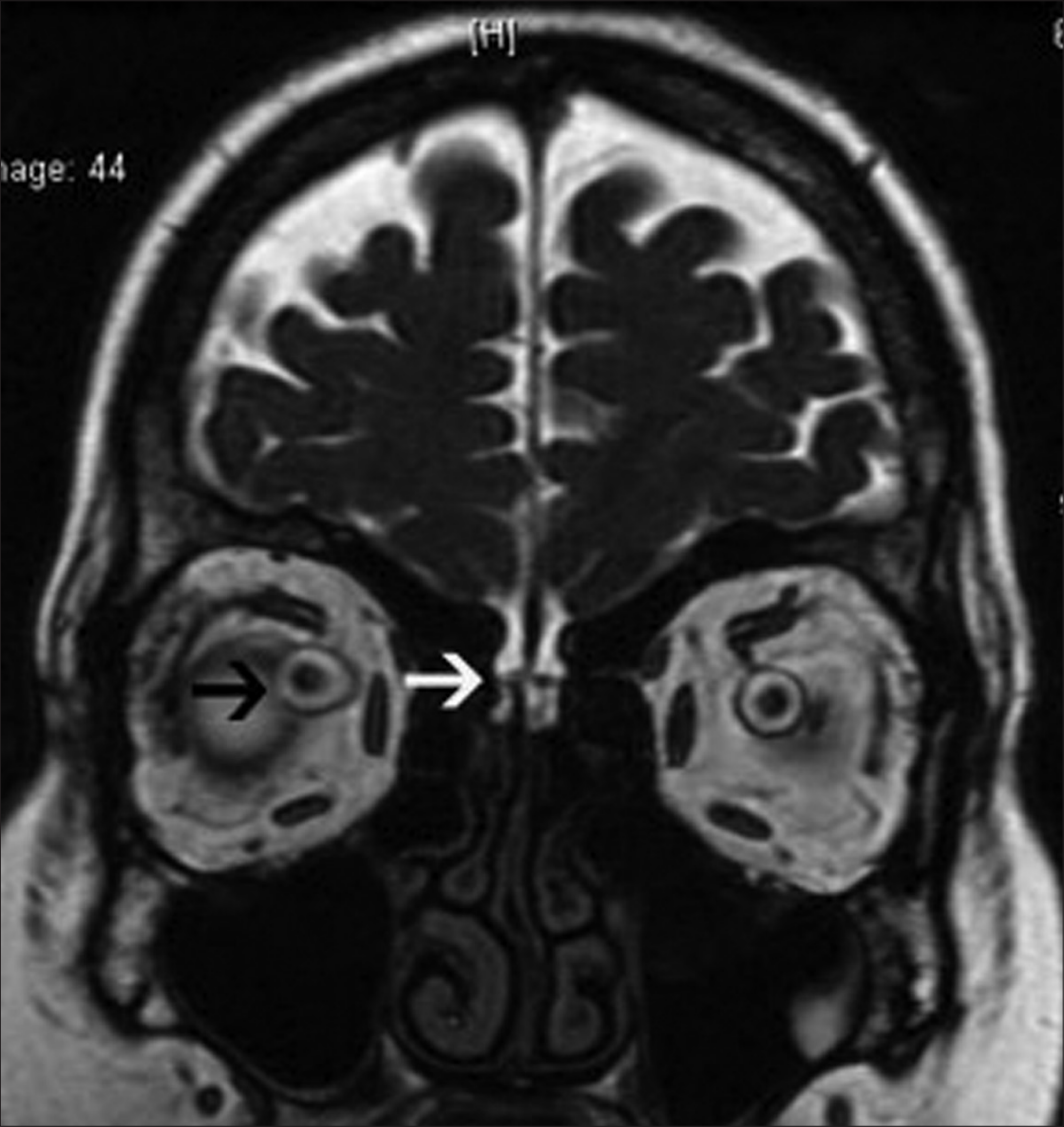
The coronal T2-weighted image reveals a leak in the cribriform plate into the anterior ethmoid cells (white arrow) and distension of the perioptic subarachnoid space (black arrow)

**Figure 2:**
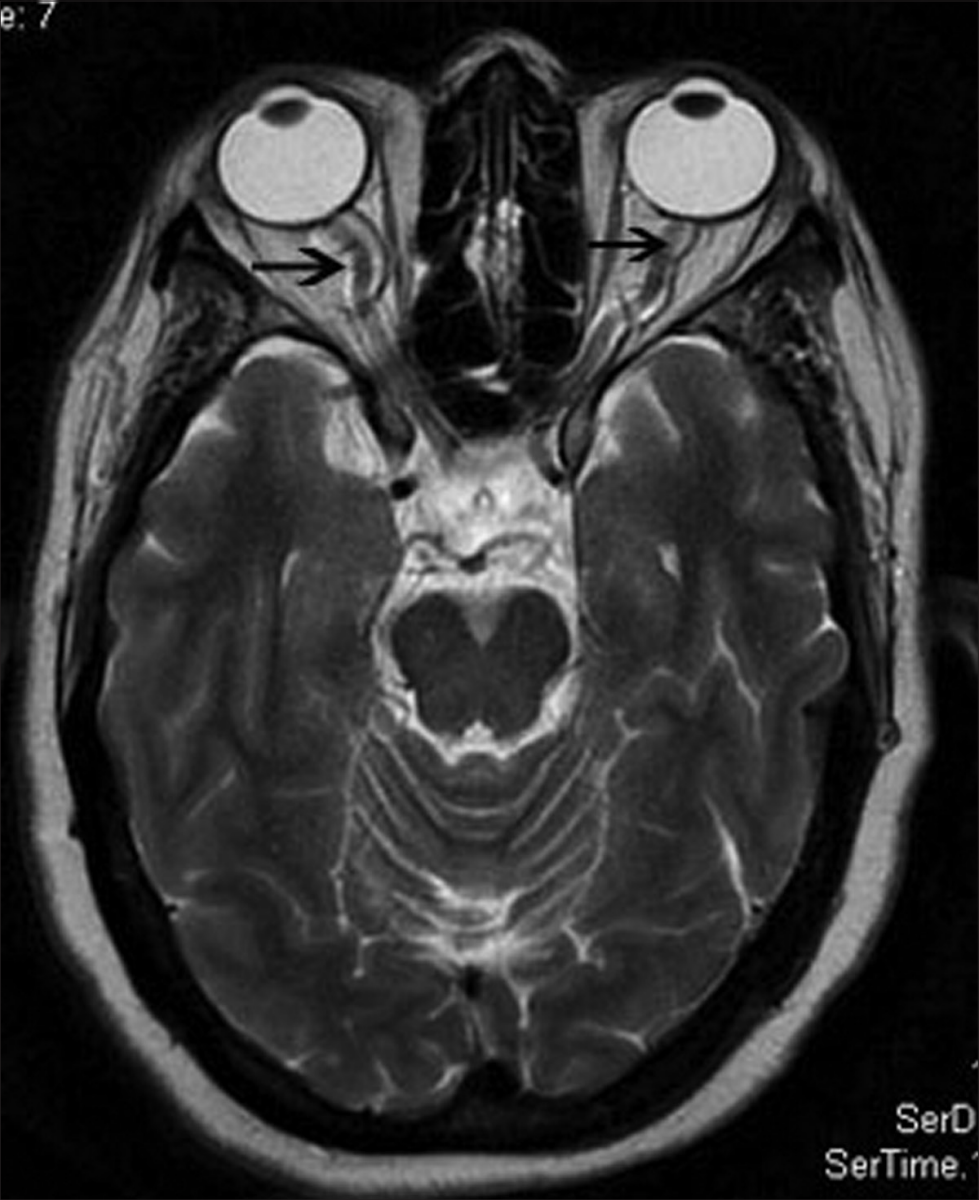
Transverse T2-weighted image shows vertical tortuosity and elongation of the optic nerve (black arrow)

**Figure 3:**
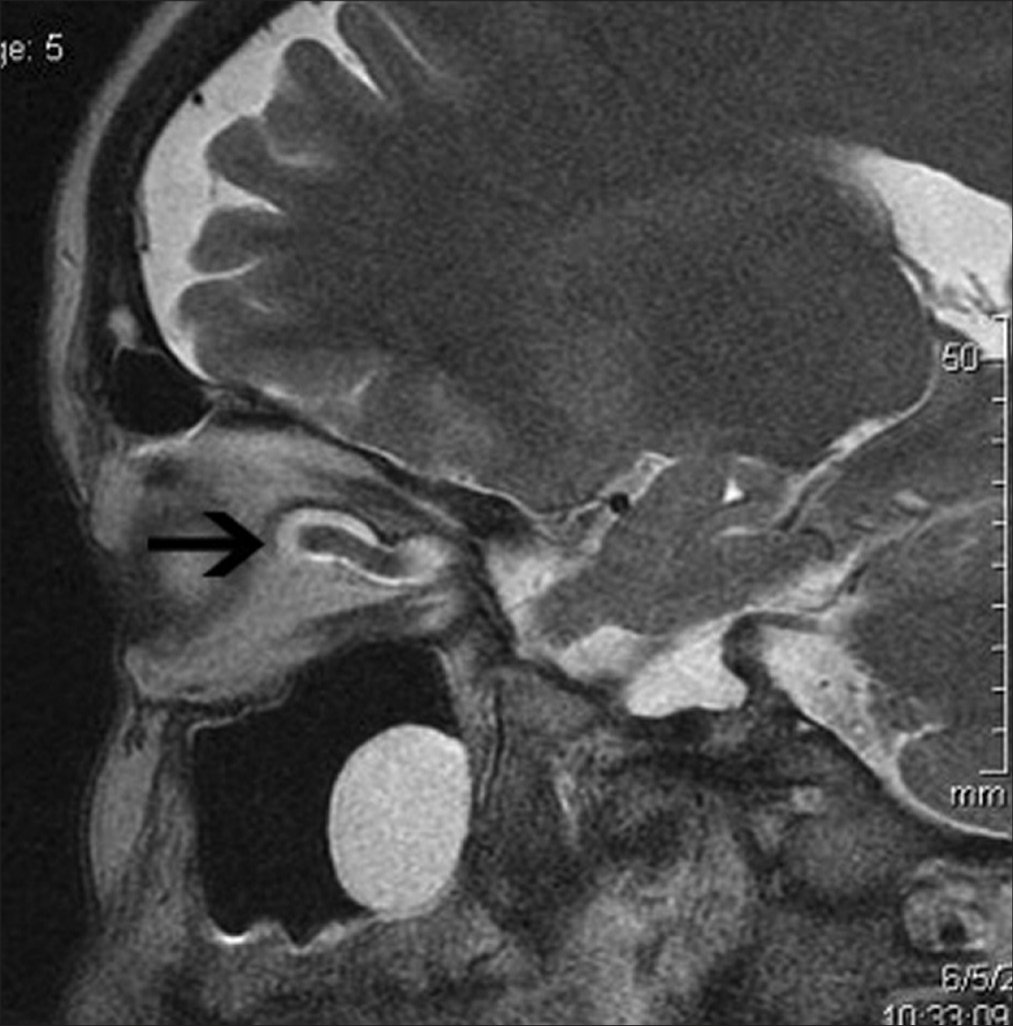
Sagittal T2-weighted image shows vertical tortuosity of optic nerve ( black arrow)

**Figure 4:**
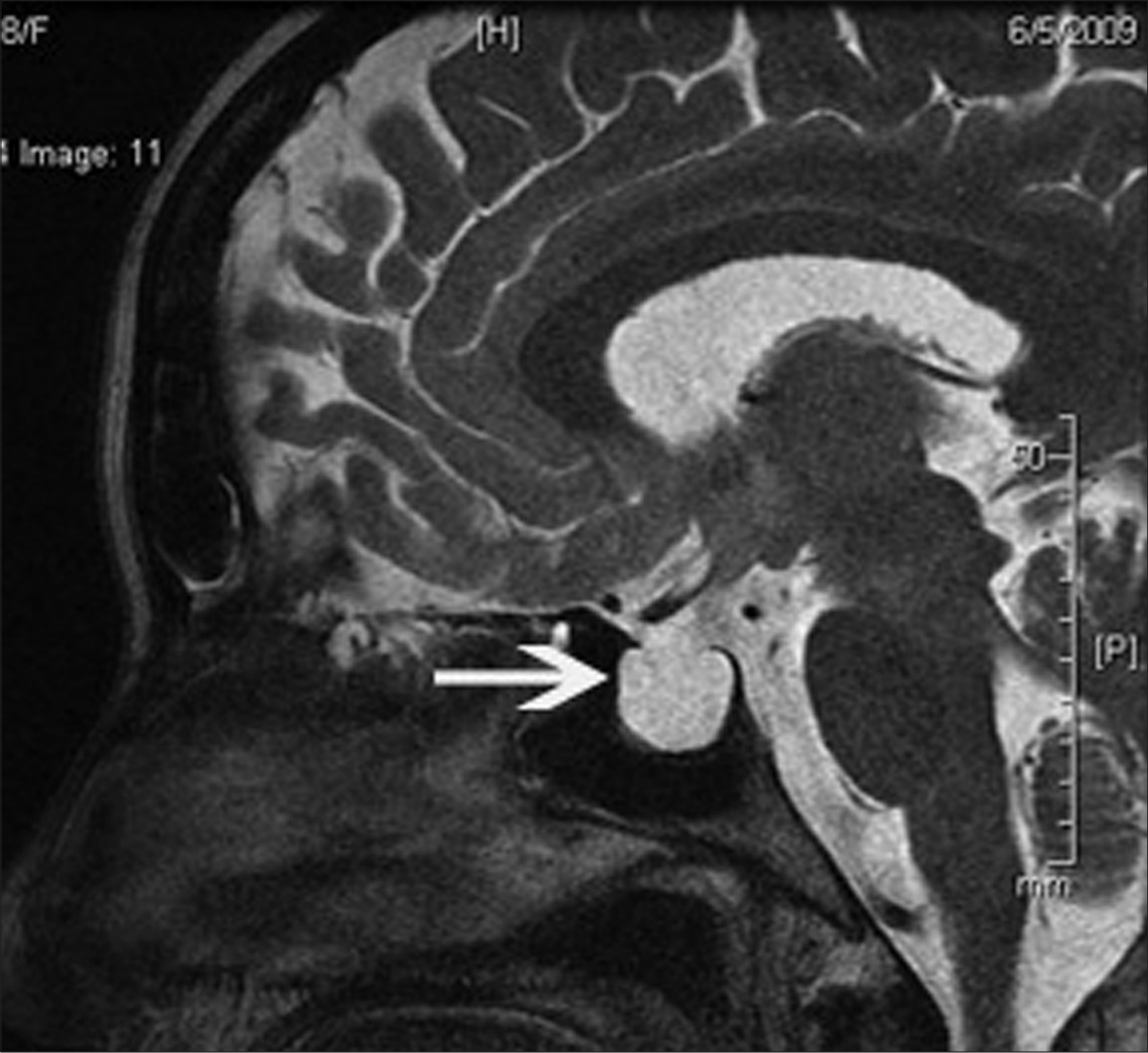
Sagittal T2-weighted image shows complete empty sella (white arrow)

A 35-year-old obese woman with body mass index of 33 kg/m^2^ presented with spontaneous intermittent CSF rhinorrhea of 6 months duration. There was history of episodic pulsatile tinnitus for the last 1 year. There was no history of headache, vomiting, visual symptom, or head injury. Neurologic examination was normal. An ophthalmologic assessment revealed normal fundus, visual acuity, and visual field (by perimetry). Magnetic resonance imaging (MRI) of brain demonstrated a leak in the cribriform plate into the anterior ethmoid cells [Figure 1]. In addition, distension of the perioptic subarachnoid space [Figure 1], elongation and vertical tortuosity of the optic nerve [Figures 2 and 3] and complete empty sella [Figure 4] were also found. MR venogram was normal. Based on these finding, a radiological diagnosis of idiopathic intracranial hypertension (IIH) was made. A lumbar puncture done 1 month later, after the resolution of CSF rhinorrhea, revealed opening pressure of 270 mm H_2_O and a normal cell count, protein, and glucose, confirming the radiological diagnosis. The patient was treated with acetazolamide and advised surgical closure but the patient denied.

IIH presenting as CSF rhinorrhea is very rare.[1] There is no mention of CSF rhinorrhea as a presenting symptom in the neurology review literature of IIH. It is probably underestimated.[2] In a series of 11 patients with spontaneous CSF leaks, CSF pressure measurements after sealing the defect, confirmed a diagnosis of IIH in 8 patients (72%).[2]

MRI finding in IIH include flattening of the posterior sclera, an empty sella, distension of the perioptic subarachnoid space, enhancement of the prelaminar optic nerve, vertical tortuosity of the orbital optic nerve, and intraocular protrusion of the prelaminar optic nerve.[3] The long-standing effect of pulsatile CSF under high pressure leads to expansion and eventual rupture of the arachnoid sleeve surrounding the olfactory filaments which pass through the pits in the cribriform plate, resulting CSF rhinorrhea.[1] Direct transmission of the elevated CSF pressure results in distension of the perioptic subarachnoid space and ballooning of the optic papilla, causing it to protrude physically into the posterior aspect of the globe.[3] The exaggerated CSF pulsatile flow also leads to downward herniation of an arachnocele through a defect in the diaphragma sella.[4]

In our case, MRI of the optic nerves and pituitary fossa provided important clues to the diagnosis of IIH. Our patient showed distension of the perioptic subarachnoid space, vertical tortuosity of the orbital optic nerve, and complete empty sella on MRI, from which a diagnosis of IIH was strongly suspected and CSF pressure measurement was therefore performed. To the best of our knowledge, there have been no reports of idiopathic intracranial hypertension presenting as CSF rhinorrhea without any other classical symptoms of IIH.

CSF opening pressure is not always measured for those with spontaneous CSF rhinorrhea, when the results of initial neuroimaging are grossly normal. Therefore, attention to the optic nerves and pituitary fossa should be given to the MRI of patients with spontaneous CSF rhinorrhea. In all suspected cases, CSF pressure should be measured and treatments of the IIH are warranted before the surgical repair of CSF leak.
